# Performance of a molecular assay to detect *Mycobacterium tuberculosis* complex DNA in clinical specimens: multicenter study in Brazil

**DOI:** 10.1590/0074-02760160196

**Published:** 2017-02

**Authors:** Mirela Verza, Karen Barros Schmid, Regina Bones Barcellos, Natali Linck, Graziele Lima Bello, Daniel Scapin, Rosa Dea Sperhacke, Márcia Susana Nunes Silva, Claudia Wollheim, Martha Gabriela Celle Rivero, Afrânio Lineu Kritski, Leonides Rezende, Martha Maria Oliveira, Elis Regina Dalla Costa, Maria Lucia Rosa Rossetti

**Affiliations:** 1 Centro de Desenvolvimento Científico e Tecnológico, Fundação Estadual de Produção e Pesquisa em Saúde, Porto Alegre, RS, Brasil; 2 Universidade de Caxias do Sul, Laboratório de Pesquisa em HIV/AIDS, Caxias do Sul, RS, Brasil; 3 Universidade de Caxias do Sul, Laboratório de Microbiologia Clínica, Caxias do Sul, RS, Brasil; 4 Universidade Federal de São Paulo, Laboratório Especial de Microbiologia Clínica, São Paulo, SP, Brasil; 5 Universidade Federal do Rio de Janeiro, Faculdade de Medicina, Centro de Pesquisa em Tuberculose do Hospital Universitário Clementino Fraga Filho, Laboratório de Genética e Biologia Molecular, Rio de Janeiro, RJ, Brasil; 6 Labtest, Belo Horizonte, MG, Brasil; 7 Universidade Luterana do Brasil, Canoas, RS, Brasil; 8 Rede Brasileira de Pesquisa em Tuberculose, Rio de Janeiro, RJ, Brasil

**Keywords:** tuberculosis, reverse-hybridisation assay, IS6110, molecular diagnosis, Detect-TB

## Abstract

**BACKGROUND:**

In high tuberculosis (TB) burden countries, there are few data on the performance of new molecular commercialised assays developed locally.

**OBJECTIVE:**

To evaluate the performance of a new molecular commercialised assay for TB diagnosis (Detect-TB) in three laboratories.

**METHODS:**

A total of 302 sputum samples from an equal number of patients with presumptive diagnosis of pulmonary tuberculosis (PTB) were submitted for routine smear microscopy, culture, and Detect-TB assay at three different sites in Brazil (the cities of Caxias do Sul, São Paulo and Canoas).

**FINDINGS:**

Seventy four (24.7%) TB cases were diagnosed (65 bacteriologically confirmed). When compared to smear microscopy/culture results, the overall sensitivity and specificity of Detect-TB assay was 84.6% (CI 95%; 73.7-91.6) and 93.1% (CI 95%; 89.1-95.8), respectively. When compared to bacteriological and clinical diagnostic criteria, the sensitivity and specificity of Detect-TB assay was 74.3% (CI 95%; 63.3-82.9) and 92.9% (CI 95%; 88.7-95.6), respectively. Among the three sites - Caxias do Sul, São Paulo and Canoas - the sensitivity and specificity were respectively 94.7% and 97.8%; 71.4% and 93.9%, 82.1% and 88.9%.

**MAIN CONCLUSIONS:**

These findings suggest that the Detect-TB assay could be applied routinely in reference laboratories across different regions in Brazil.

The early and accurate diagnosis of tuberculosis (TB) remains a crucial step in the management and control of TB. The World Health Organization (WHO) estimates that one third of all TB cases remains undiagnosed (WHO 2015). Despite the advance in TB diagnosis, with the introduction of new molecular tests, the difficulties of an early detection of the cases remains a major challenge.

In 2014, the Brazilian Ministry of Health, following WHO recommendations, implemented the molecular semi-automated test Xpert MTB/RIF (Cepheid Inc., Sunnyvale, California) in 94 of the priority cities to control TB, in order to substitute smear microscopy of patients with presumed diagnosis of pulmonary TB (MS/SVS 2015). This assay provides detection of TB and rifampin resistance directly from untreated sputum in less than 2 h. The implementation of Xpert MTB/RIF in nine countries under programmatic conditions evaluated by Creswel et al. (2014), verified an increase of 30% to 50% in the number of confirmed TB cases, but there was no detectable increase in the number of reported cases. Similar results were found in a study performed in Brazil with 14 laboratories in two cities (Durovni et al. 2014). However, this test remains costly for developing nations despite subsidies, as it requires a constant power supply, and involves at least one annual calibration performed by a trained technician.

Other commercial tests, such as AccuProbe (Gen-Probe, USA) (O’Donnell et al. 2012) and GenoType MTBDRPlus (Hain Life science, Germany) (Maschmann et al. 2013), are reliable, but too expensive for routine use in most low-resource countries. For these reasons, new molecular commercialised assays for TB diagnosis locally developed may be useful and more cost effective.

Our group has previously developed and evaluated an *in-house* polymerase chain reaction (PCR) using *IS*6110 as a target and tested three different procedures to detect *Mycobacterium tuberculosis* complex DNA: agarose gel electrophoresis, membrane hybridisation and microwell plate hybridisation (Rossetti et al. 1997, Sperhacke et al. 2004, Verza et al. 2009, Maschmann et al. 2011, Michelon et al. 2011). Based on the microwell plate hybridisation, the *in-house* procedure has evolved to a molecular kit, the Detect-TB assay that has emerged to cooperate with smear microscopy on TB diagnosis.

The current study describes the performance evaluation of a commercial molecular assay, Detect-TB, developed by LabTest, Belo Horizonte, Brazil, which detects the *M. tuberculosis* complex in respiratory clinical specimens. Taking into account the different disease incidence rates and laboratory characteristics, we have carried out a multicenter study in three Brazilian cities.

## MATERIALS AND METHODS

*Study sites* - The multicenter study was carried out in three reference laboratories located in different cities in Brazil from January 2011 to December 2012. The aim was to evaluate the performance of commercial Detect-TB assay compared to reference standard methods. The following laboratories were included: (a) Laboratório Especial de Microbiologia Clínica (LEMC), Universidade Federal de São Paulo (UNIFESP), São Paulo city, in the Southeast Region; (b) Laboratório de Microbiologia Clínica (LMC), Universidade de Caxias do Sul (UCS), Caxias do Sul city, in the South Region; and (c) Laboratório de Biologia Molecular (LBM), Universidade Luterana do Brasil, Canoas city, in the South Region. These sites were chosen for their different incidence rates of 54.2, 22.0 and 94.2 of TB per 100,000 inhabitants, respectively. LEMC-UNIFESP is a routine and research laboratory, and evaluates clinical samples from patients admitted to the University Hospital in São Paulo, LMC-UCS is a reference laboratory for clinical evaluations in Caxias do Sul, and LBM-ULBRA is a research laboratory that analyses clinical samples from Health Units in Canoas city.

*Specimens and patients* - 302 sputum samples (one sample per patient), on the basis of routine testing, from presumed pulmonary TB patients without primary TB treatment in the past, were collected to estimate the accuracy of the Detect-TB assay: 42 clinical specimens from São Paulo, 112 from Caxias do Sul, and 148 from Canoas. Mycobacterial cultures and smear microscopy were carried out for all clinical samples. Bias was minimised through blinding in which the technicians performing the molecular and reference tests were unaware of the other test results.

The sputum specimens were treated with Ziehl-Neelsen staining and culture on Löwenstein-Jensen medium and identification of *M. tuberculosis* complex (MTBC) with biochemical tests, according to MS/SVS recommendations (MS/SVS 2008).

*Procedure* - The method, based on Michelon et al. (2011), was developed by our group at the Centro de Desenvolvimento Científico e Tecnológico (CDCT), Fundação Estadual de Produção e Pesquisa em Saúde (FEPPS), Porto Alegre, Brazil, and patented at INPI (Instituto Nacional da Propriedade Industrial) under number PI 0900612-5. It began to be commercialised after registration at ANVISA with number 10009010259, named Detect-TB assay.

Briefly, *M. tuberculosis* (MTB)-complex DNA was amplified using biotinylated primers targeting the *IS*6110 fragment. These amplified products were reverse-hybridised on microwell plates (Nunc Immobilizer™ Amino Surface, Nunc A/S, Roskilde, Denmark) to a fixed aminated probe complementary to the internal region of the amplified *IS*6110 fragment. The hybridisation signal was detected by colorimetry using the streptavidin-peroxidase/TMB system and measured using a spectophotometer with a 450/620 nm filter. The absorbance of the negative control was subtracted from the results and all samples with readings above 0.275 were considered positive for MTB complex DNA, as recommended by Detect-TB (Michelon et al. 2011).

*Study design* - To evaluate the assay at different rates of TB incidence and geographical locations, Detect-TB assays were sent to the three study sites cited above. All laboratory personnel involved in the study received a detailed protocol and underwent a one-week training course. To facilitate the reproducibility of the results, all tests were performed with commercialised assays from the same production lot, thereby ensuring that the tests used contained reagents from batches that had been produced previously and tested in accordance to the current Brazilian specific law (good manufacturing practices for the control of diagnostic products - ANVISA- decree 686, 08.27.1998).

Bacteriologically confirmed pulmonary tuberculosis (PTB) cases were defined when the patient matched at least one of two of the following criteria: (a) two smear positive for acid-fast bacilli; or (b) *M. tuberculosis* (Mtb) culture-positive in respiratory specimens. Non-bacteriologically confirmed PTB cases were defined as those that had clinical and radiological diagnosis and clinical improvement after six months of solely anti-TB treatment. Non-TB cases were defined as those that did not receive anti-TB treatment for the following two years, according to the Brazilian National Disease Reporting System (SINAN).

*Statistical analysis* - Detect-TB assay performance was compared to reference standard methods: TB cases with bacteriologically confirmed criteria and those with composite criteria that include TB cases with and without bacteriologically confirmation. Sensitivity, specificity, accuracy and kappa tests were calculated using the Statistical Package for Social Sciences (SPSS) v16.0. P-values lower than 0.05 were considered statistically significant.

*Ethics* - This study was approved by the Ethical Committee of Fundação Estadual de Produção e Pesquisa em Saúde do Rio Grande do Sul (FEPPS-RS) under the number 01/2007.

## RESULTS

The cut-off value of the assay (0.250) was defined previously by Michelon et al. (2011). The gray zone was defined as the values from 0.250 to 0.275. Overall, among 302 clinical samples, only 3 (0.99%) had results in the gray zone. All samples in the gray zone were repeated and the results remained inconclusive. For this reason, these samples were removed from the analysis.

A total of 299 sputum samples were evaluated, 74 (24.7%) TB cases were diagnosed of which 65 were bacteriologically confirmed (Table). When compared to bacteriological confirmed diagnosis, the overall sensitivity and specificity of Detect-TB assay was 84.6% (CI 95%; 73.7-91.6) and 93.1% (CI 95%; 89.1-95.8), respectively. When compared to composite diagnosis criteria, the sensitivity and specificity of Detect-TB assay was 74.3% (CI 95%; 63.3-82.9) and 92.9% (CI 95%; 88.7-95.6), respectively. Median turnaround time from sputum collection to the release of Detect-TB assay results was two days.

**TABLE:** Detect-TB results compared to laboratory and clinical methods for the sites involved in the multicenter study

Bacteriological diagnosis	Canoas					Caxias do Sul					São Paulo				

	N	Detect-TB	False negative	False positive	Gray zone	N	Detect-TB	False negative	False positive	Gray zone	N	Detect-TB	False negative	False positive	Gray zone
Positive	39	32	7		-	19	18	1	-	-	7	5	2	-	-
Smear Pos/cult Neg	3	2	1	-	-	2	2	-	-	-	-	-	-	-	-
Smear Neg/cult Pos	16	10	6	-	-	1	-	1	-	-	1	-	1	-	-
Smear Pos/cult Pos	20	20	-	-	-	16	16	-	-	-	6	5	1	-	-
Negative	109	97	-	12	-	93	90	-	2	1	35	31	-	2	2

Total	148	131	7	12	-	112	108	1	2	1	42	30	2	2	2

Sensitivity (%; CI 95%)	82.1 (67.0-91.0)					94.7 (73.5-99.9)					71.4 (35.2-92.4)				
Specificity (%; CI 95%)	88.9 (81.8-93.7)					97.8 (91.9-99.8)					93.9 (79.4-99.3)				
Kappa	0.682					0.907					0.654				
Clinical Diagnosis															
Positive	41	32	9	-	-	20	18	2	-	-	13	5	8	-	-
Negative	107	95	-	12	-	92	89	-	2	1	29	25	-	2	2

Total	148	127	9	12	-	112	107	2	2	1	42	30	8	2	2

Sensitivity (%; CI 95%)	78.1 (63.1-88.2)					90.0 (68.7-98.4)					38.5 (17.6-64.6)				
Specificity (%; CI 95%)	90.5 (83.2-94.9)					97.8 (91.8-99.8)					92.6 (75.5-99.1)				
Kappa	0.654					0.878					0.353				

CI: confidence intervals; TB: tuberculosis.

In Canoas city, 39 of the 148 analysed samples had positive results using smear microscopy or culture methods. Detect-TB assay showed a sensitivity of 82.1% (CI 95%; 67.0-91.0), specificity of 88.9% (CI 95%; 81.8-93.7), and a kappa value of 0.682 (CI 95%; 0.611-0.766) (Table). According to composite diagnosis criteria, of the 148 samples from Canoas, 41 had positive results. Detect-TB assay sensitivity, specificity kappa value were of 78.1% (CI 95%; 63.1-88.2), 90.5% (CI 95%; 83.2-94.9) and of 0.654 (CI 95%; 0.573-0.734), respectively (Table).

At the Caxias do Sul site, 19 of the 112 samples had positive results using smear microscopy and/or culture methods. Detect-TB assay showed sensitivity of 94.7% (CI 95%; 90.0-99.3), specificity of 97.8% (CI 95%; 94.6-100.0), and a kappa value of 0.907 (CI 95%; 0.848-0.965) (Table). According to composite diagnostic criteria, 20 of the 112 samples tested were positive for TB. Detect-TB assay showed sensitivity of 90.0% (CI 95%; 83.9-96.0), specificity of 97.8% (CI 95%; 94.6-100.0), and a kappa value of 0.878 (CI 95%; 0.812-0.943) (Table).

In São Paulo city, seven of the 42 analysed samples had positive results using smear microscopy or culture methods. Detect-TB assay showed sensitivity of 71.4% (CI 95%; 56.1-86.6), specificity of 93.9% (CI 95%; 85.2-102.0), and a kappa value of 0.654 (CI 95%; 0.494-0.813) (Table). According to composite diagnostic criteria, 13 out of 42 samples at the São Paulo site tested positive. Detect-TB assay showed a sensitivity of 38.5% (CI 95%; 22.1-54.8), specificity of 92.6% (CI 95%; 83.2-101.0), and a kappa value of 0.353 (CI 95%; 0.192-0.513) (Table).

## DISCUSSION

A multicenter study was carried out for the performance evaluation of the Detect-TB assay for the detection of *M. tuberculosis* complex DNA in clinical samples. For detection, amplified products with the expected size of 245 bp (*IS*6110) were reverse-hybridised to a microwell plate. This test was selected for its simple method and colorimetric end point, factors that facilitate the adoption of new nucleic acid amplification tests at laboratories at least in middle income countries that can provide laboratories with molecular techniques (Dowdy et al. 2003, van Cleeff et al. 2005).

At the three sites (Canoas, Caxias do Sul and São Paulo cities), when compared to confirmed bacteriological diagnosis, the overall sensitivity of the Detect-TB assay ranged from 82.1 to 97.8% and the specificity ranged from 71.4% to 94.7%. When compared to composite diagnostic criteria, sensitivity ranged from 38.5% to 90.0% and specificity ranged from 88.7% to 97.8%. The results obtained at these sites are in agreement with the findings from other studies and with commercialised assays (Piersimoni & Scarparo 2003, Ling et al. 2008, Michelon et al. 2011, O’Donnell et al. 2012, Bhutia et al. 2013, Steingart et al. 2013). The rate of false-positive results in all sites were of 2.2% at Caxias do Sul, 6.9% at Sao Paulo and 8.1% at Canoas.

Detect-TB is an assay to be performed in research and/or clinical laboratories according to the manufacturer’s instructions. As any other molecular technique, good laboratory practices must be followed, including unidirectional workflow, different rooms for specimen separation and processing, and reagent preparation. The use of a negative control in all steps of the protocol, from DNA extraction to DNA amplification detection, ensures the identification of contamination event during the analysis. However, this handling procedure occurs at post sample separation. The higher rate of false positive results observed in Canoas city (8.1%) compared to the other sites could be explained due to cross-contamination that could have occurred during specimen processing. Although we followed all the guidelines for diagnostic methods, before the molecular analysis, at the Hospital Laboratory, the samples may have been manipulated in the same cabinet that the solid culture was performed, and this could have led to sputum contamination, which can only be detected at the molecular level. Generally, the results of Detect-TB assay showed better specificity with composite diagnostic criteria when compared to laboratory criteria. In a previous study by our group, we demonstrated that smear microscopy plus Detect-TB assay was the most cost-effective initial diagnostic test for active TB when applied in a prison population with a high prevalence of active PTB, indicating that Detect-TB assay has great potential to be implemented under routine diagnostic conditions (Schmid et al. 2014). The overall rate of false negatives was higher when the Detect-TB assay was to composite diagnostic criteria (6.3%) than with bacteriological criteria (3.3%). A high number of false-negative samples was found in São Paulo (19.0% with clinical diagnosis and 4.7% with laboratory diagnosis). This observation is most likely related to the characteristics of the TB patients evaluated in a university hospital, which is a tertiary care unit that is not the reference for TB treatment. The patients assisted at this level of care are usually more paucibacillary, as reported by others (Michelon et al. 2011). The incorrect processing of the samples during homogenisation and decontamination could also have led to unequal aliquots during processing for smear microscopy, culture and PCR. This phenomenon may have resulted in an insufficient number of detectable bacilli that were suitable only for detection by smear or culture. In this case, an inappropriate sample dilution during the decontamination procedure or a sampling error could have reduced the sensitivity of the test, generating a false-negative result as described elsewhere (Woods 2001, Piersimoni & Scarparo 2003, Chakravorty et al. 2005). The Detect-TB assay has proved sensitive to the detection of smear microscopy negative samples (Michelon et al. 2011). In this study, Detect-TB assay was able to detect four cases of smear microscopy positive with culture negative samples, probably due to the presence of non-viable bacteria, and notably detected 10 cases out of 16 of smear microscopy negative with culture positive samples. In this scenario, Detect-TB assay results were released in only two days, lower than the turnaround time related to the culture results (~30 days). Depending on the prevalence of active TB in the population, nucleic acid amplification taken along with the full clinical picture could be useful to rull in or rull out the anti-TB treatment. The nucleic acid amplification test result is only one piece of evidence and would not alone define a clinical decision. One limitation of our study might rely on the assumption that we considered as TB cases those with two positive smear microscopy and culture negative. More than one sputum positive smear microscopy result supports the diagnosis of mycobacterial respiratory disease but does not allow differentiation of TB from non-mycobacterial disease. Additionally, it is uncommon to have non-mycobacterial diseases with two positive smear microscopy. In our sample it occurred in only three cases. Therefore, we can infer that those cases had a high probability to be active TB cases instead a non-mycobacterial disease.

There is a high degree of variability across studies in the accuracy of nucleic acid amplification tests. Assuming the TB prevalence at each site and the accuracy based upon the final clinical criteria plus laboratory confirmed diagnosis of PTB observed at the three different laboratories (Table), high negative predictive values (94% in Canoas, 89% in Caxias and 95% in São Paulo) and moderate positive predictive values (71% in Canoas, 89% in Caxias and 70% in São Paulo) were obtained for all sites. Those figures are also achieved even with Xpert MTB/RIF. Assuming that Xpert MTB/RIF has a sensitivity of 90% and specificity of 98%, having HIV negative presumed TB subjects evaluated at health services with TB prevalence of 1% at primary health care centers, 10% at emergency units and 20% at hospitals or reference ambulatory centers, the negative predictive values achieved with this test were respectively 99.8%, 98.8% and 97.5%, and positive predictive values 31.0%, 83.0%, 92.0%. Smear microscopy and culture techniques remain important diagnostic techniques to be used in addition to Xpert MTB/RIF, in regions with low TB prevalence, in order to increase the positive predictive value.

Therefore, molecular tests cannot replace culture but should be used in addition to conventional tests and clinical data for TB diagnosis as highlighted by others (Pai et al. 2003, 2004, Piersimoni & Scarparo 2003, CDC 2009). Furthermore, the Detect-TB assay is similar to an ELISA as it requires common laboratory equipment to reference laboratories from middle income countries with a high burden of TB and HIV, and it has the advantage of being a low test cost (US$ 10,00), when compared to other current molecular commercialised assays. In addition, the assay results, which are based on spectrophotometry, are independent of human interpretation, instead of visual interpretation colorimetric assays (Kox et al. 1996).
